# Risk stratification of patients with familial hypercholesterolemia in a multi-ethnic cohort

**DOI:** 10.1186/1476-511X-13-65

**Published:** 2014-04-08

**Authors:** Matthew D Allard, Ramesh Saeedi, Masoud Yousefi, Jiri Frohlich

**Affiliations:** 1 Healthy Heart Program Prevention Clinic, St Paul’s Hospital, Vancouver, University of British Columbia, Vancouver, Canada; 2 Pathology and Laboratory Medicine, University of British Columbia, St. Paul’s Hospital, Rm 180 - 1081 Burrard Street, Vancouver, BC V6Z 1Y6, Canada; 3 Department of Medicine, University of British Columbia, Vancouver, Canada

**Keywords:** Familial hypercholesterolemia, Cardiovascular disease (CVD), Risk factors, Gender differences and ethnicity

## Abstract

**Background:**

Heterozygous Familial hypercholesterolemia (FH) is a common autosomal dominant disorder resulting in in very high blood cholesterol levels and premature cardiovascular disease (CVD). However, there is a wide variation in the occurrence of CVD in these patients. The aim of this study is to determine risk factors that are responsible for the variability of CVD events in FH patients.

**Methods:**

This is a retrospective analysis of a large multiethnic cohort of patients with definite FH attending the Healthy Heart Prevention Clinic in Vancouver, Canada. Cox proportional hazard regression analysis was used to assess the association of the risk factors to the hard cardiovascular outcomes.

**Results:**

409 patients were identified as having “definite” FH, according to the Dutch Lipid Clinic Network Criteria (DLCNC), with 111 (27%) having evidence of CVD. Male sex, family history of premature CVD, diabetes mellitus, low high density lipoprotein cholesterol (HDL-C) and high lipoprotein (a) (Lp (a)) were significant, independent risk factors for CVD. In men, family history, diabetes and low levels of HDL-C were significant risk factors while in women smoking, diabetes mellitus and high Lp (a) were significant risk factors for CVD. There were no significant differences in risk factors between ethnicities.

**Conclusion:**

In conclusion, men and women differ in the impact of the risk factors on the presence of CVD with family history of CVD and low HDL-C being a significant factor in men while smoking and increased Lp (a) were significant factors in women. Diabetes was a significant factor in both men and women.

## Background

Heterozygous familial hypercholesterolemia (FH) is an autosomal dominant disease with a prevalence of 1:500 in the general population. Major causes of FH are loss-of-function mutations in the low-density lipoprotein (LDL) receptor (LDL-R) or apolipoprotein B-100 (apo B) gene, or gain-of-function mutations in proprotein convertase subtilisin/kexin type 9 (PCSK9), resulting in very high blood cholesterol levels and premature CVD [1,2]. If left untreated, it is estimated that about 50% of men and 12% of women will suffer a coronary episode before the age of 50 [1,2]. FH has been shown to decrease one’s life expectancy by approximately 20-30 years [1,2]. The variation in phenotypic expression, and specifically the occurrence of CVD in FH is affected by contributions of additional metabolic, environmental and genetic risk factors, acting in conjunction with severe hypercholesterolemia. Treatment of FH with lipid lowering therapy has proven to be both highly effective and cost efficient [3,4]. Despite the proven benefits of early diagnosis and treatment of FH, only a minority of patients are diagnosed and treated adequately and the majority remains untreated or improperly treated at the present time. Both lay people and health professionals currently lack full awareness of FH, its diagnostic features, and its consequences. Moreover, standard assessment tools (eg. Framingham) do not accurately quantify risk in these patients. Thus, a better means of identifying and characterizing risk in these patients is required. The aim of this study was to investigate the effects of risk factors that may contribute to CVD in FH patients.

## Results

### Characteristics of study population

Four hundred and nine patients were identified as having “definite” FH as determined by the DLCNC (Table 1). There were more women (229) than men (180). 111 (27%) patients had hard evidence of CVD with more men affected (58.6%). The mean age of onset of CVD was 49.5 (±12.4) in men and 56.9 (±16.0) in women. Coronary artery disease (n = 92, 82.8%), cerebrovascular disease (n = 9, 7.8%), peripheral ischemic disease (n = 4, 3.4%) or aortic valve disease (n = 6, 6.0%) were the underlying pathologies. According to data obtained from BC Vital Statistics, 24 patients (12 men, 12 women) died. 13 of these patients (52.0%) died of a cardiovascular event.

**Table 1:** Dutch lipid clinic network criteria

		**Score**
**Family history**		
First degree relative with known premature coronary and vascular disease (Men <55 yrs, Females <60 yrs),		1
OR		
First degree relative with LDL cholesterol above the 95^th^ percentile for age and sex		
First degree relative with tendionous xanthomata and/or arcus cornealis,		2
OR		
Children <18 yrs with LDL cholesterol above the 95^th^ percentile for age and sex		
**Clinical history**		
Patient with premature coronary artery disease (ages as above)		2
Patient with premature cerebral or peripheral vascular disease (ages as above)		1
**Physical examination**		
Tendinous xanthomata		6
Arcus cornealis prior to age 45 yrs		4
LDL cholesterol (mmol/L)		
	LDL-C ≥ 8.5	8
	LDL-C 6.5-8.4	5
	LDL-C 5.0-6.4	3
	LDL-C 4.0-4.9	1
DNA analysis- functional mutation in the *LDLR*, *APOB*, or *PCSK9* gene		8
**Stratification**		**Total score**
Definite FH		≥8
Probable FH		6-7
Possible FH		3-5
Unlikely FH		<3

Modified from Civeira [3] with permission from Elsevier.

Demographics and clinical characteristics of the patients with and without CVD are shown in Tables 2 and 3. Patients with CVD were older, men and smokers, with a higher prevalence of hypertension, diabetes mellitus and family history of premature CVD. HDL-C levels were significantly lower in patients with CVD. Paradoxically, the LDL-C levels were significantly higher in the non-CVD group. We speculate that this may have resulted from several factors including referral bias, lifestyle changes, higher Lp (a) levels or different size of LDL particles in the two groups. Also, patients with CVD tended to have higher Lp (a) (349 mg L^-1^ (IQR: 122.0,841.2) vs. 262.6 mg L^-1^( IQR: 104.4,605.1)). Demographics and clinical characteristics of the patients with and without CVD (only those untreated) are shown in Tables 4 and 5. The same significant differences remain except for family history of premature CVD.

**Table 2:** Characteristics of the entire cohort stratified by presence or absence of cardiovascular disease

**Risk factor**		**With CVD**	**Without CVD**	**P-value**
		**N = 111**	**N = 298**	
Age (years)		66.6(±13.5)	58.6(±15.2)	<0.001
Mean (SD)				
Age at 1^st^ CVD endpoint (yrs)		52.6(±14.6)	NA	NA
Mean (SD)				
Sex (M/F,%)		60.4/39.6	38.6/61.4	<0.001
BMI (kg/m^2^)		26.2(±4.5)	26.1(±4.9)	0.456
Mean (SD)				
Diabetes (%)		13.5	3.7	0.001
Family history (%)				0.019
No family history of pre-mature CVD		39.5	49.8	
Family history of CVD; pre-mature		60.5	50.2	
Smoking (%)				<0.001
	Never smoked	39.6	63.4	
	Ever smoked	60.4	36.6	
Hypertension (%)		34.2	18.5	0.001
Deaths (%)		15.3	2.3	<0.001

**Table 3:** Laboratory results of the entire cohort at a patient’s first clinic visit

**Risk factor**	**With CVD**	**Without CVD**	**P-value**
	**N = 111**	**N = 298**	
Total cholesterol (mmol/L)	7.8(2.1)	8.7(2.0)	<0.001
Mean (SD)			
HDL cholesterol (mmol/L)	1.2(0.4)	1.3(0.4)	0.001
Mean (SD)			
LDL-cholesterol (mmol/L)	5.7(2.0)	6.6(1.9)	<0.001
Mean (SD)			
Triglycerides (mmol/L)	1.6(0.9)	1.7(1.2)	0.509
Mean (SD)			
TC/HDL	7.1(3.5)	7.0(2.7)	0.724
Mean (SD)			
Lp (a) (mg/L)*	349.0	262.6	0.166
Median (IQR)	(122.0,841.2)	(104.4,605.1)	
Fasting blood sugar (mmol/L)	5.4(0.6)	5.1(0.5)	0.001
Mean (SD)			
Lipid lowering therapy (%)	33.3	25.8	0.139

*:Lp (a) analysis carried out on the 84 FH patients with CVD and Lp (a) data available and the 200 FH patients with no CVD and Lp (a) data available.

**Table 4:** Characteristics of the entire cohort stratified by presence or absence of cardiovascular disease (only untreated patients)

**Risk factor**		**With CVD**	**Without CVD**	**P-value**
		**N = 74**	**N = 221**	
Age (years)		67.7 (±13.3)	58.0 (±15.8)	<0.001
Mean (SD)				
Age at 1^st^ CVD endpoint (yrs)		53.6 (±14.5)	NA	NA
Mean (SD)				
Sex (M/F,%)		58.1/41.9	37.6/62.4	<0.001
BMI (kg/m^2^)		26.2 (±4.6)	25.6 (±4.8)	0.355
Mean (SD)				
Diabetes (%)		13.5	3.6	0.001
Family history (%)				0.420
No family history of pre-mature CVD		45.9	51.6	
Family history of CVD; pre-mature		54.1	47.5	
Smoking (%)				<0.001
	Never smoked	41.9	65.2	
	Ever smoked	58.1	34.8	
Hypertension (%)		32.4	16.3	0.001
Deaths (%)		16.2	2.7	<0.001

**Table 5:** Laboratory results of the entire cohort at a patient’s first clinic visit (only untreated patients)

**Risk factor**	**With CVD**	**Without CVD**	**P-value**
	**N = 74**	**N = 221**	
Total cholesterol (mmol/L)	8.2(1.8)	9.1(2.1)	<0.001
Mean (SD)			
HDL cholesterol (mmol/L)	1.2(0.3)	1.4(0.4)	0.001
Mean (SD)			
LDL-cholesterol (mmol/L)	6.1(1.8)	7.0(1.9)	<0.001
Mean (SD)			
Triglycerides (mmol/L)	1.7(0.9)	1.7(1.2)	0.968
Mean (SD)			
TC/HDL	7.5(3.5)	7.3(2.8)	0.684
Mean (SD)			
Lp (a) (mg/L)*	331.5	278.8	0.480
Median (IQR)	(111.2,691.6)	(102.6,569.7)	
Fasting blood sugar (mmol/L)	5.4(0.6)	5.1(0.5)	0.001
Mean (SD)			
Lipid lowering therapy (%)	0.0	0.0	NA

Females tended to have higher Lp (a) (298 mg L^-1^ (IQR: 129.0, 709.8) vs. 264 mg L^-1^(IQR: 88.8, 594.8)) than men. Men with CVD were younger (64 ± 13 vs. 71 ± 13) and had their first CVD event 7 years earlier than women with CVD (50 vs. 57). Men with CVD had significantly lower HDL-C levels (1.1 mmol/L (0.3) vs. 1.4 mmol/L (0.4)) and significantly higher TC/HDL ratio (7.8 ± 4.0 vs. 6.2 ± 2.4), while, women with CVD had significantly higher median Lp (a) levels (512 mg L^-1^ (IQR: 163, 925) vs. 289 mg L^-1^ (IQR: 87, 604)) than men.

The median (interquartile range) was 288.2 (IQR:109.679.9) mg L^-1^, and there was no significant difference in the median Lp (a) concentration between men and women (*P* = 0.36, Mann–Whitney test). There were 205 patients whose Lp (a) concentration was categorized as low (<600 mg L^-1^) and 79 as high (>600 mg L^-1^). Of these, 55 patients (26.8%) in the low group had a first CVD event in the follow-up period compared with 29 (36.7%) in the high group. The mean age at the first CVD event was 53.9 years (range, 23–84 years) in the cohort as a whole: 54.8 years in the low Lp (a) group (range, 23– 84 years) and 52.3 years (range, 32–73 years) in the high Lp (a) group (*P* = 0.59).

### Relation between CVD and risk factors

Table 6 shows the relative risks and 95% confidence intervals for the risk factors in univariate and multivariate analyses for the entire cohort. In both univariate and multivariate Cox hazard analysis, male sex, premature family history of CVD, diabetes mellitus and high Lp (a) all significantly increased CVD risk, while higher levels of HDL-C at first visit decreased risk for CVD. Smoking only significantly increased CVD risk in univariate analysis. In a paradoxical finding LDL-C at first visit appeared to decrease risk for CVD. We speculate that this have resulted from several factors including, referral bias, lifestyle changes, higher Lp (a) levels or different size of LDL particles in the two groups.

**Table 6:** Relative risks (RR) and 95% confidence intervals (95% CI) for the presence of a risk factor for cardiovascular disease in the entire cohort

	**Univariate**			**Multivariate (n = 409)**		
	**RR**	**95% CI**	**P value**	**RR**	**95% CI**	**P value**
Diabetes (present vs. absent)	3.2	1.9-5.6	<0.001	3.6	2.0-6.5	<0.001
Male sex	2.9	2.0-4.3	<0.001	2.4	1.6-3.7	<0.001
Lp (a) (high vs. low)*	1.6	1.0-2.6	0.033	1.8	1.1-2.9	0.014
HDL-C (mmol L^-1^)	0.2	0.1-0.4	<0.001	0.4	0.2-0.7	0.004
Family history pre-mature CVD	1.5	1.1-2.3	0.026	1.8	1.2-2.7	0.005
LDL-C (mmol L^-1^)	0.8	0.6-0.9	0.002	0.8	0.7-0.9	0.001
Smoking (ever vs. never)	2.1	1.4-3.0	<0.001	-	-	-

*:Lp (a) analysis carried out on the 276 FH patients with Lp (a) data available.

Table 7 shows the relative risks and 95% confidence intervals for the risk factors in univariate and multivariate analyses for the entire cohort without anyone on treatment at baseline. The same significant results are seen as in the entire cohort except for family history of premature CVD and high Lp (a) which were not significant in either univariate or multivariate analysis.

**Table 7:** Relative risks (RR) and 95% confidence intervals (95% CI) for the presence of a risk factor for cardiovascular disease in the entire cohort (only untreated patients)

	**Univariate**			**Multivariate (n = 295)**		
	**RR**	**95% CI**	**P value**	**RR**	**95% CI**	**P value**
Diabetes (present vs. absent)	3.8	2.0-7.1	<0.001	3.8	2.0-7.5	<0.001
Male sex	2.7	1.7-4.3	<0.001	1.9	1.1-3.2	0.018
Lp (a) (high vs. low)*	-	-	-	-	-	-
HDL-C (mmol L^-1^)	0.2	0.1-0.4	<0.001	0.3	0.1-0.7	0.008
Family history pre-mature CVD	-	-	-	-	-	-
LDL-C (mmol L^-1^)	0.8	0.7-0.9	0.001	0.8	0.6-0.9	0.002
Smoking (ever vs. never)	1.8	1.1-2.9	0.014	-	-	-

*:Lp (a) analysis carried out on the 199 untreated FH patients with Lp (a) data available.

Table 8 provides the relative risks and 95% confidence intervals of the risk factors in univariate and multivariate analyses stratified by sex. In men, in univariate Cox hazard regression analysis, diabetes mellitus, and premature family history of CVD significantly increased CVD risk, while HDL-C at first visit decreased risk for CVD. In multivariate Cox hazard regression analysis family history and diabetes mellitus were significant risk factors for CVD, while HDL-C at first visit decreased risk for CVD. In women, in univariate Cox hazard regression analysis, diabetes mellitus, high Lp (a) and smoking, significantly increased CVD risk. In multivariate Cox hazard regression analysis, diabetes mellitus, high Lp (a), and smoking were significant risk factors for CVD.

**Table 8:** Relative risks (RR) and 95% confidence intervals (95% CI) for the presence of a risk factor for cardiovascular disease in men and women

	**Univariate**			**Multivariate (n, men = 180, n, women = 229)**		
	**RR**	**95% CI**	**P value**	**RR**	**95% CI**	**P value**
Diabetes (present vs. absent)						
Men	2.7	1.1-6.3	0.024	3.1	1.2-8.0	0.018
Women	5.4	2.5-11.6	<0.001	4.3	2.0-9.4	<0.001
Family history pre-mature CVD						
Men	2.0	1.2-3.2	0.009	2.1	1.3-3.6	0.004
Women	1.4	0.8-2.6	0.235	-	-	-
HDL-C (mmol L^-1^)						
Men	0.3	0.1-0.8	0.014	0.3	0.1-0.8	0.022
Women	0.5	0.2-1.1	0.066	-	-	-
Smoking (ever vs. never)						
Men	1.2	0.7-2.0	0.441	-	-	-
Women	2.9	1.6-5.3	0.001	2.5	1.4-4.7	0.003
Lp (a) (high vs. low)*						
Men	1.1	0.5-2.2	0.814	-	-	-
Women	2.8	1.4-5.3	0.002	2.8	1.4-5.3	0.003

*:Lp (a) analysis carried out on the 123 male FH patients with Lp (a) data available and 161 female FH patients with Lp (a) data.

## Discussion

We assessed the contribution of risk factors to the development of CVD in a large, multi-ethnic cohort of FH patients to identify those most susceptible to the development of CVD. Our study confirms earlier identified associations by a number of other similar FH cohort studies (9-17). Risk factors found to be significant, in decreasing order of importance, were male sex, diabetes, high Lp (a), family history of pre-mature CVD, and low HDL-C. Of note is that hypertension, a well-known risk factor in the general population, was not an independent risk factor in our study.

Although a number of the risk factors explored using the Cox multivariate survival analysis were significant within the entire cohort, of particular interest were the few risk factors that were sex specific. For instance, family history of pre-mature CVD was a significant risk factor of CVD only in men with a relative risk of 2.1 (95% CI, 1.3-3.6). Despite family history of premature CVD being an established independent risk factor for CVD and atherosclerosis in the general population, no similar FH cohort studies have explored it as a risk factor. We found it to be a significant and independent risk factor for CVD in our entire cohort but interestingly it was significant only in men. It is likely a combination of the family’s lifestyle (diet, smoking, physical activity) and the inherited genetic predisposition that contribute to the increased risk in those with a family history of premature CVD. A study by de Jong et al. [5] found that endothelial function, a predictor of future cardiovascular events, was impaired in children with FH having family history of premature cardiovascular events. Additionally, HDL-C, with a relative risk of 0.3 (95% CI, 0.1-0.8) was of significance only in men.

In contrast, smoking, with a relative risk of 2.5 (95% CI, 1.4-4.7) and Lp (a), with a relative risk of 2.8 (95% CI, 1.4-5.3), were of significance only in women. These results suggest that there are certain risk factors more detrimental to one sex and identifying their presence can identify an FH patient more susceptible to developing CVD.

In accordance with Holmes et al. [6] the median Lp (a) levels in this FH cohort were significantly higher than in patients without FH. Interestingly, in our study median Lp (a) levels between men and women with FH when comparing Lp (a) values within our total FH study cohort were similar. However, when comparing Lp (a) levels between women with CVD to men with CVD, the women’s Lp (a) level was significantly higher. This is consistent with our previous findings [7]. In accordance with this finding, Lp (a) level was found to be a significant risk factor in women but not in men. A study by Nenseter et al. [8] of Lp (a) in CVD resistant vs. CVD susceptible FH patients similarly found that Lp (a) levels were more important in women. They found that CVD-susceptible women had significantly higher levels of Lp (a) compared to CVD-resistant women, and CVD-resistant women had significantly lower Lp (a) levels compared to CVD-resistant men [8]. The findings of our study and those by Nenseter et al., suggest that Lp (a) is a more important risk factor in women.

Despite the introduction of statins, 50% of mortality in this cohort was due to CVD- almost twice as much as expected in the general population.

In contrast to other FH cohort studies [9-17], we studied an ethnically heterogeneous population of FH patients. Thus, these results are more applicable to the type of FH patients seen in lipid clinics across North America. We used only CVD endpoints that were unambiguous such as myocardial infarction (MI), coronary artery bypass graft surgery (CABG), percutaneous coronary transluminal angioplasty (PCTA)/ stent, stroke, carotid endartarectomy, femoral-popliteal bypass, cardiac death, aortic valve replacement and positive coronary angiogram with greater than 50% occlusion. This allowed for avoidance of any endpoints that might be open to interpretation such as angina, transient ischemic attack (TIA), nuclear medicine tests, and exercise stress tests. Although this certainly limited the number of subjects defined as having CVD, it makes the results more meaningful.

The present study has a number of limitations mainly due to its retrospective nature. We relied on the accuracy of the physician’s written record. Other FH studies recorded lipid levels in patients who were not using lipid lowering therapy for a period of time whereas, due to the retrospective nature of data collection, we recorded lipid values at a patient’s first visit. The patients in the CVD group were being more aggressively treated, thus, confounding the lipid values we recorded. Due to this, the non-CVD group had a less optimal lipid panel than the CVD group. There was also a recruitment bias present in this study as a result of the manner we chose to diagnose patients with FH. Genetic information/diagnosis was unavailable on most patients and as a result we used clinical criteria to diagnose FH. We limited our cohort those diagnosed as “Definite FH” based upon the DLCNC. Also there may have been a selection bias as the cohort of patients consisted of those referred to the lipid clinic.

## Conclusion

In conclusion, in our ethnically diverse cohort of FH patients the significant risk factors for CVD in decreasing order of importance were, male sex, diabetes, high Lp (a), family history of pre-mature CVD, and low HDL-C. Men and women differed in the impact of the individual risk factors on the development of CVD.

## Methods

### Patients and study design

This is a retrospective cohort study using data from the Healthy Heart Prevention Clinic at St. Paul’s Hospital. Patients included in the study were anyone who visited the clinic since 1970 and were 18 years or older and diagnosed as “Definite” FH based upon the DLCNC (Table 1). Each patient included in the study signed “consent for research form” in his or her chart in accordance with hospital and/or government regulations. Patients excluded from the study were those aged less than 18 yrs, those with a diagnosis of homozygous FH, those with secondary causes of dyslipidemia, including hypothyroidism, liver disease, renal disease, HIV-induced dyslipidemia, and drug-induced dyslipidemia. Other patients excluded from the study were those who were seen at the clinic only once. The patients were followed at the lipid clinic for a minimum of 6 months and a maximum of 28 years. A detailed phenotypic characterization was completed using a designed template. The study protocol was approved by the UBC Committee of Medical Ethics.

The demographic and clinical characteristics including age, sex, family history of pre-mature CVD, body mass index (BMI), smoking, diabetes, and hypertension were recorded. Family history of pre-mature CVD was defined as the presence of a hard cardiovascular endpoint in a male 1^st^ degree relative younger than 55 or a female 1^st^ degree relative younger than 60. Smoking history was defined dichotomously as “ever smoked” or “never smoked”. Hypertension was defined as a blood pressure reading greater than 140/90 mmHg that was confirmed at a follow up appointment or patient’s treatment history. Diabetes mellitus was defined as fasting blood glucose readings greater than 7.0 mmol/L on 2 separate occasions and/or hemoglobulin A1C greater or equal than 6.5% or patient’s treatment history.

The baseline fasting lipid profile was recorded TC, HDL-C, TG and calculated LDL-C using the Freidewald formula. Lp (a) levels were also recorded. Prior to 2005 Lp (a) levels were measured using an immunometric immunoassay ((reference range (RR) < 400 U/L). After 2005 Lp (a) levels were measured using the commercially available direct-binding double mAb-based method (Mercodia Lp (a) ELISA) (RR < 300 mg/L). The results are expressed in mg/dl, where 1 U of apo (a) is approximately equal to 0.7 mg of Lp (a) (Mercodia Mannual). Due to the retrospective nature of the study approximately 28% of subjects did not have an untreated lipid profile. The individuals in the CVD group have been more aggressively treated and as a result their TC and LDL-C are significantly lower than those in the non-CVD group. To account for this the same analyses were performed on the cohort on no lipid lowering medication at baseline.

### CVD endpoints

Cardiovascular endpoints were MI, CABG, PCTA stent, stroke, angiogram with >50% occlusion, carotid endarterectomy, femoral popliteal bypass, aortic valve replacement and cardiac death. Both the type and year of the first CVD endpoint were recorded.

### Statistical analyses

All statistical analyses were performed using the SPSS package (Version 21.0). For normally distributed continuous characteristics data were presented as means and standard deviations. The continuous clinical characteristics with skewed distributions were presented as medians and interquartile ranges. Categorical variables were presented as numbers with corresponding percentages. Differences in clinical characteristics between patients with and without CVD were tested with chi-square statistics for categorical variables, the non-parametric test Mann Whitney U Test for Lp (a) and triglycerides, and t-tests for the normally distributed continuous characteristics.

Cox proportional hazard regression analysis was used to assess the association of risk factors to hard cardiovascular outcomes in univariate and multivariate analyses. Follow-up started from the date of birth and ended at the date of first CVD event. Patients without CVD were censored at the date of the last lipid clinic visit or at the date of death attributable to other causes.

The following variables were entered into the analyses: sex, BMI, smoking history, family history of pre-mature CVD, diabetes, hypertension, LDL-C at first visit, HDL-C at first visit, TG at first visit, and Lp (a) (very high: >600 mg/L or low: <75^th^ percentile, <600 mg/L).

## Abbreviations

FH: Familial hypercholesterolemia; CVD: Cardiovascular disease; DLCNC: Dutch lipid clinic network criteria; HDL-C: High density lipoprotein cholesterol; Lp (a): Lipoprotein (a); LDL: Low density lipoprotein; LDL-R: Low density lipoprotein receptor; Apo-B: Apolipoprotein B100; PCSK9: Proprotein convertase subtilin/kexin 9; TG: Triglycerides; MI: Myocardial infarction; CABG: Coronary artery bypass graft; PCTA: Percutaneous transluminal angioplasty; TIA: Transient ischemic attack; BMI: Body mass index.

## Competing interests

The authors declared that they have no competing interest.

## Authors’ contributions

MA reviewed patient’s medical charts to collect the data, helped carry out statistical analysis and drafted the manuscript. RS participated in the design and coordination of the study and helped to draft the manuscript. MY carried out statistical analysis. JF conceived of the study, and participated in its design and coordination and helped to draft the manuscript. All authors read and approved the final manuscript.
